# Modulating the tumor immune microenvironment with locoregional image-guided interventions

**DOI:** 10.3389/fimmu.2022.1057597

**Published:** 2023-01-04

**Authors:** Samagra Jain, Rahul A. Sheth

**Affiliations:** ^1^ Department of Radiology, Baylor College of Medicine, Houston, TX, United States; ^2^ Department of Interventional Radiology, MD Anderson Cancer Center, Houston, TX, United States

**Keywords:** immunotherapy, image guidance, interventional radiography, cancer, transarterial administration

## Abstract

Cancer immunotherapy has gained significant attention in recent years and has revolutionized the modern approach to cancer therapy. However, cancer immunotherapy is still limited in its full potential due to various tumor immune-avoidance behaviors and delivery barriers, and this is seen in the low objective response rates of most cancers to immunotherapy. A novel approach to immunotherapy utilizes image-guided administration of immunotherapeutic agents directly into a tumor site; this technique offers several advantages, including avoidance of potent toxicity, bypassing the tumor immunosuppressive microenvironment, and higher therapeutic bioavailability relative to systemic drug administration. This review presents the biological rationale for locoregional image-guided immunotherapy administration, summarizes the existing interventional oncology approaches to immunotherapy, and discusses emerging technological advances in biomaterials and drug delivery that could further advance the field of interventional oncology.

## Introduction

Immunotherapy with immune checkpoint inhibitors has revolutionized cancer care by stimulating the body’s adaptive immune system to detect, engage, and eliminate cancerous cells (1). Recent advances in basic science have identified immunosuppressive immune checkpoints such as Cytotoxic T-lymphocyte–Associated Protein 4 (CTLA-4), Programmed Cell Death Protein 1 (PD-1), and Programmed Cell Death Ligand 1 (PD-L1), enabling the development of clinically viable immunotherapy strategies by blocking or bypassing these inhibitory signals (2). Several immunotherapy-based regimens have received FDA approval for applications across the cancer spectrum including multiple myeloma, melanoma, colorectal cancer, endometrial carcinoma, and diffuse large B-cell lymphoma (3). However, cancer immunotherapy is still limited in its clinical applications due to several factors including the immunosuppressive tumor microenvironment, poor intratumoral infiltration of therapeutics, and T cell exhaustion, along with systemic toxicity and poor drug accumulation in tumor sites (4). Indeed, the overall response rate for the majority of cancer types remains less than 40% (5).

The field of interventional radiology is well-poised to drive innovation in cancer immunotherapy. Image-guided interventions have already been used to establish several novel and minimally invasive approaches to cancer treatment, including radiofrequency/thermal tumor ablation, trans-arterial chemoembolization, and targeted delivery of yttrium-90 (Y-90) emitting microspheres (6). These treatments often have fewer systemic side effects than conventional chemotherapeutics with similar tumor reduction potential due to their targeted delivery to tumor cells, and it stands to reason that highly precise immunotherapy delivery can similarly be enabled with image-guided approaches. Recent preclinical studies have established the therapeutic viability of locoregional immunotherapy delivery, demonstrating significant potential to revolutionize the modern approach to cancer treatment (7). Furthermore, complementary advances in biomaterials and targeted drug delivery technologies can further increase the selectivity and localization of immunotherapy to cancerous tissue. Engineered methods such as nanoparticle encapsulation of therapeutics, magnetic resonance targeting, and clustered regularly interspaced short palindromic repeats (CRISPR) – CRIPSR associated protein 9 (Cas9) multiplex editing systems can be combined with image-guided injections for robust and highly precise administration of cancer therapeutics. This review summarizes the biological underpinnings of cancer immunotherapy, presents existing approaches by the field of interventional oncology in this space, and discusses translational technologies that will play an important role in future treatment strategies.

### Cancer immunotherapy

The basis of cancer immunotherapy lies in transforming the adaptive immune system to be able to recognize and eliminate previously unrecognizable tumor tissue, resulting in sustained antitumor activity from the body’s natural immune mechanisms. Current approaches utilize immunotherapy alone as well as in conjunction with conventional chemotherapeutics/radiation depending on the type and stage of tumor (1). In a typical immune response from diseased tissue, damage-associated molecular patterns (DAMPs) are released from dying cells in the form of proteins, cytokines, or other biological molecules. These are detected by pattern recognition receptors (PRRs) on dendritic cells (also known as antigen presenting cells or APCs), which then activate cytotoxic T lymphocytes (CTLs) with the CD8+ surface marker. The main effector cell of the adaptive immune system, CD8+ T cells are specifically aimed at the antigen conveyed by the dendritic cells and will eliminate cells around the body expressing it with a combination of membrane perforation and protease activity (8).

A significant barrier to this natural process is the ability of tumor cells to evade detection by the immune system and downregulate the cellular immune response. Various mechanisms are utilized by cancers to prevent immunogenicity, ranging from intrinsic regions of hypoxia and elevated lactate levels to production of immunosuppressive cytokines that directly deactivate T cell responses (9). Additionally, if APCs are unable to phagocytose tumor antigens, then T cell remain inactivated; tumors can take advantage of this fact by producing neoantigens that are unable to be efficiently processed by the APCs. Certain protein-protein interactions between APCs and T cells known as immune checkpoints are also used to physiologically suppress immune activation; well-known examples of protein receptors on T cells include CTLA-4 and PD-1/PD-L1 (10). Many modern immunotherapy approaches revolve around bypassing these suppressive mechanisms with strategies such as inducing cancer cell apoptosis to generate immune-recognizable DAMPs or blocking immune checkpoint inhibitors to prevent immune downregulation (11). Chimeric antigen receptor (CAR) therapy, a technique which involves re-engineering an individual’s T cells with a cancer targeting receptor, has also successfully been employed against certain leukemias and lymphomas (12). These therapies are far from perfect – ongoing clinical data shows limited response to cancers and only in a certain populations, and the metabolic toxicity associated with immunotherapy continues to remain high. T cell localization and penetration in larger or avascular cancers can also be difficult to achieve, and the immunosuppressive microenvironment continues to limit the clinical efficacy of these therapies (4).

## Existing interventional oncology immunotherapy approaches

### Rationale for locoregional immunotherapy

**Figure 1:**
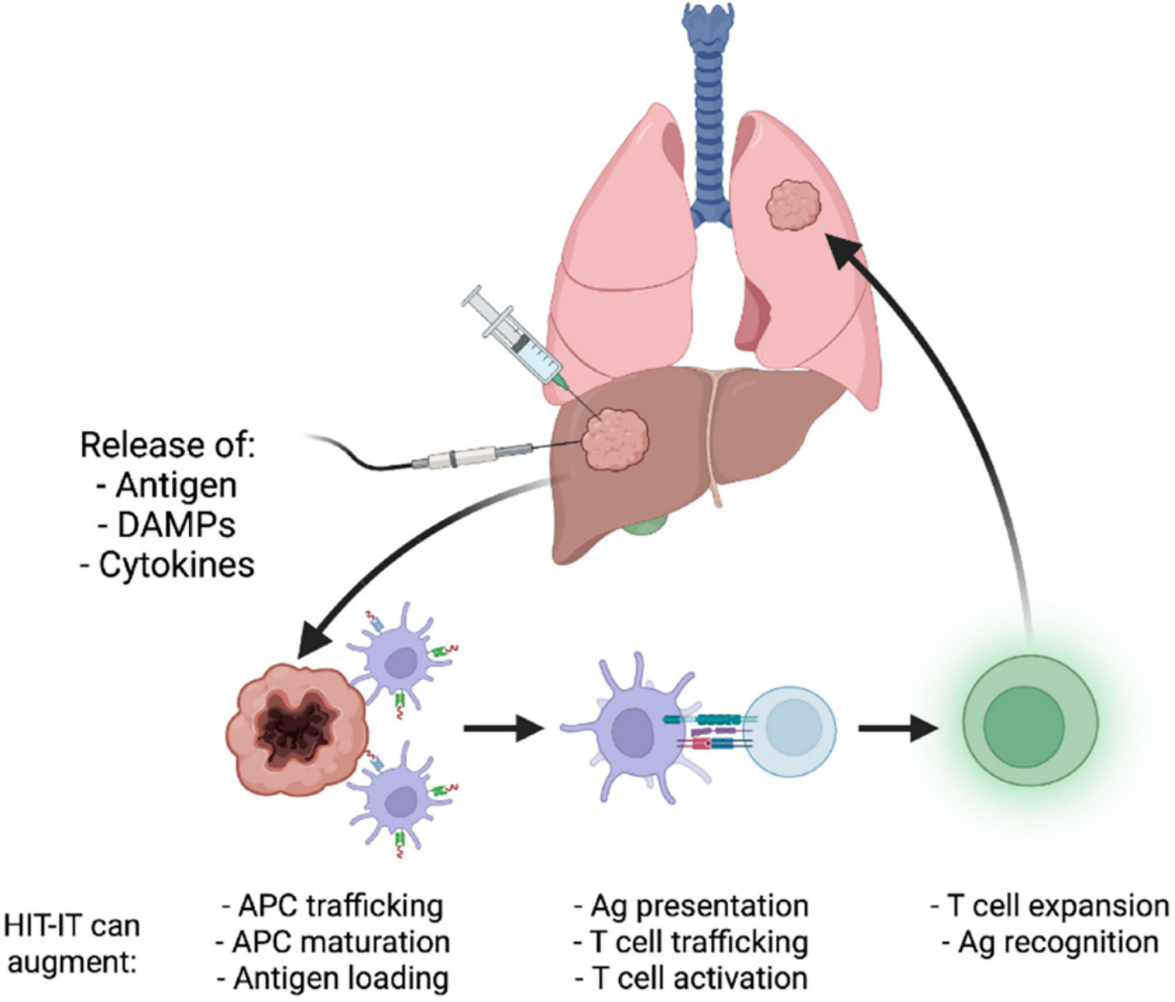
Illustration of the mechanism underlying intratumoral cancer immunotherapy. Reproduced without change from Senders et. al. (17) (HIT-IT = human intratumoral immunotherapy, Ag, antigen).

Locoregional delivery of immunotherapy utilizing interventional oncology approaches can avoid many of the obstacles associated with current immunotherapy approaches. Using image guidance from X-ray fluoroscopy, ultrasound, magnetic resonance imaging, or computed tomography, an interventional radiologist can utilize a vascular or percutaneous approach to a tumor site and selectively administer immunotherapy directly within a tumor. One significant benefit to this approach is limited off-target effects/systemic toxicity as the therapeutic remains localized within the tumor site. This also allows for lower therapeutic doses to be delivered while concurrently achieving a higher drug concentration within tumors. Another major advantage is the ability to effectively handle metastatic lesions by individual targeting of tumors and immune system potentiation, potentially sparing the patient from systemic effects (13). The objective response rates to certain systematically administered immunotherapy continue to remain low, highlighting the need for the specific and potent response that locoregional immune modulation can provide (14). Despite the low overall response rates seen with the current use of intratumoral administration of immunotherapy, early trials for combination and monotherapy treatments point to promising outcomes in terms of patient survival. For example, phase 1 trials for glioblastoma treated with a TLR agonist administered intratumorally resulted in a medial overall survival of 7.2 months (15). Another phase 1 trial of a combination therapy of a TLR agonist with an anti-PD-1 antibody used to treat unresectable malignant melanoma saw a 12 month progression-free survival of 88% with an overall survival rate of 89% (16). It must be emphasized that phase 1 trial results cannot be generalized to clinical success (clinically translated intratumoral immunotherapy still has low response rates as mentioned earlier), but these early results are indicative of the exciting potential that intratumoral immunotherapy administration holds. **Figure 1** provides a visual depiction of the mechanism of intratumoral immunotherapy.

### Tumor microenvironment modulation

As previously described, the immunosuppressive tumor microenvironment poses a significant challenge to both traditional anticancer and novel immunotherapy approaches, so the key goal of immuno-oncology remains to induce the immune system despite these barriers. Tumor ablation and the resulting immune activation from the necrosis-associated DAMPs has proven to be a viable mechanism for this strategy and several techniques are already widely utilized in clinical practice by interventional radiologists to cause tumor immunogenic cell death. Endovascular therapies such as trans-arterial chemoembolization (TACE) and trans-arterial radioembolization (TARE), percutaneous methods including radiofrequency ablation (RFA) and cryoablation, and stereotactic laser-guided approaches have demonstrated tumor volume reduction with consequent immune activation, and immunotherapy can be utilized similarly alongside these techniques for synergistic therapeutic effects (18).

### Oncolytic viruses

Oncolytic virotherapy is a novel technique that re-engineers human viruses for cancer destruction and DAMP production. The drug talimogene laherparepvec (TVEC, trade name Imlygic) is the first and at present only FDA approved intratumoral immunotherapy and is used to treat advanced melanoma. TVEC utilizes an attenuated herpes simplex virus, type 1 (HSV-1) which has been engineered to produce granulocyte-monocyte colony stimulating factor (GM-CSF); the double stranded DNA virus can directly and preferentially lyse tumor cells, producing DAMPs and activating the previously latent adaptive immune system. TVEC has demonstrated a well-tolerated and durable response rate in a 2015 Phase III clinical trial, although 5-year survival remained low with monotherapy (19). Clinical trials are currently ongoing that are examining combination therapies of TVEC with checkpoint inhibitors, kinase inhibitors, and conventional chemoradiation therapy for melanoma and non-melanoma malignancies (NCT04068181, NCT04330430) (20, 21).

Additionally, several other viruses have been identified to have oncolytic properties, including adenovirus, poliovirus, measles virus, and coxsackievirus; though these therapeutics are still in early clinical trials, they have demonstrated oncolytic ability in preclinical models and could open up new therapeutic avenues against a wide range of malignancies (22). Notably, researchers at Duke University are conducting a Phase II clinical trial (NCT01491893) with a recombinant poliovirus/rhinovirus for use against malignant gliomas. The virus, delivered intratumorally using an intracerebral catheter into the enhancing portions of the tumor, was well tolerated in the Phase I trials by pediatric patients (23).

Systemic administration is the conventional approach for oncolytic virus delivery but therapeutic outcomes against solid tumors continue to be modest (24). The main obstacle in delivery of oncolytic viruses is the presence of neutralizing antibodies or complement that can prevent the virus from achieving oncolysis, in addition to common immunotherapy barriers such as tumor heterogeneity and immunosuppressive microenvironments. Route of administration plays an important role in overcoming these barriers; intratumoral delivery allows for a higher bioavailability but intravenous delivery can achieve a systemic response, so the unique tumor characteristics and staging should guide the approach. Other strategies to avoid immune destruction that have shown success include administration of complement inhibitors, immunosuppressants, or serotype switching (25). Additionally, novel technologies such as nanoparticles are being explored for drug delivery; encapsulation within nanosystems may be able to protect therapeutics from degradation and neutralization until they reach a target site (26).

### Intratumoral delivery of checkpoint inhibitors

Checkpoint inhibitors have proven to be one of the most transformative advances in modern immunotherapy. The immunosuppressive checkpoints mentioned earlier (CTLA-4, PD-1/PD-L1) can be blocked by therapeutics to allow for a more potent immune response, and several drugs have received FDA approval for use against various solid and hematologic malignancies (27). Pembrolizumab is a prototypical PD-1 antagonist that blocks the PD-1 receptor from activation by PD-L1, and ipilimumab serves a similar function against the CTLA-4 receptor. As revolutionary as these therapies have been, these are systemic immunotherapies that are currently administered intravenously and encounter many of the associated problems including systemic toxicities and low bioavailability (28). Intratumoral administration of checkpoint inhibitors in preclinical models has been shown to increase T cell antitumor activity as well as avoid systemic symptoms. Notably, a 2013 study in mice models demonstrated that subcutaneous slow-release of CTLA-4 blocking antibodies with a lipid adjuvant could induce local and systemic antitumor activity without systemic side effects. Additionally, a far lower dose of antibody was required to see similar effects as systemic administration (29). Most current clinical trials are centered on combining locoregional therapies (TACE, TARE) with systemic administration of immunomodulators (NCT04975932), but checkpoint inhibitors have significant potential for monotherapy *via* intratumoral delivery as well.

### Innate immune system activation

Much of the discussion around cancer immunotherapy has been centered on induction of adaptive immunity, but the innate immune system can be a potent and synergistic mediator of antitumor activity and especially so in tumors with limited T cell infiltration. In addition to the previously discussed dendritic cells, macrophages and natural killer (NK) cells can contribute to tumor elimination by phagocytosis and cytotoxic mechanisms. The innate immune system is especially sensitive to external antigens, with dedicated sensing pathways for bacterial toxins (Toll-Like Receptors, or TLRs) and nucleic acids *via* the cyclic GMP–AMP synthase (cGAS) – stimulator of interferon genes (STING) pathway. Activation of these pathways promotes transcription of pro-inflammatory cytokines and interferons which can allow for more potent antitumor responses (30). Therapeutic formulations utilizing synthetic TLR activation *via* intratumoral injection have already received FDA approval against bladder cancer and basal cell carcinoma, and several early phase clinical trials utilizing combination therapies of TLR agonists and checkpoint inhibitors have shown strong promise against melanoma and squamous cell carcinoma (NCT02668770) (31). Similarly, the cGAS/STING pathway, an innate mechanism responsible for pathogenic detecting cytosolic DNA, can be exploited with synthetic cyclic dinucleotides (CDNs). Clinical trials are also underway to evaluate the antitumor activity of cGAS/STING agonist formulations as monotherapy or as adjuvants (NCT05321940) (32). However, chronic inflammatory states can provide a favorable environment for tumorigenesis and metastasis; this complication must be considered in the development of innate immune system agonists.

### Intratumoral delivery of CAR-T cells

CAR-T cells, which are T cells engineered for anti-cancer activity, have demonstrated significant activity against various types of hematological malignancies. Multiple formulations of systemic CAR-T cell therapeutics have received FDA approval for use against B-cell acute lymphocytic leukemia (B-ALL), non-Hodgkin lymphoma (NHL), and multiple myeloma (33). However, this same potency is not observed against solid tumors – response rates are under 9% – due to physiological barriers including T cell exhaustion, poor intratumoral penetration, and tumor antigen heterogeneity (34). Additionally, systemic administration of CAR-T cells has several associated risks, including cytokine release syndrome, neurological effects/encephalopathy, and coagulopathy (35). Recently, intratumoral administration of CAR-T cells has begun to be explored as a potential avenue for increasing efficacy against solid tumors. For example, a 2022 study by Ghosn et al. explored the feasibility of image-guided intrapleural CAR-T cell administration, finding no adverse events in patients with pleural malignancy (36). Additionally, earlier studies have established the ability of intratumoral CAR-T cells to evoke an inflammatory response and cause tumor necrosis in breast tissue specifically, which future studies will hopefully continue to build upon (37). The efficacy and response rates of intratumoral CAR-T cell delivery against the current standard of care cannot be stated with confidence yet, with most studies still in early Phase 1 clinical trials (NCT04951141); however, this technique has significant potential implications for the treatment of liver, neurological, breast and many other solid tumor malignancies.

## Novel technologies in immunotherapy

Nanoparticle-mediated drug delivery is rapidly gaining popularity in clinical applications and may offer solutions to delivery limitations imposed by the immunosuppressive tumor microenvironment. Nanoparticles are defined as materials with a size range between 1 and 100 nm and can be synthesized from a variety of materials, including lipids, polysaccharides, and inorganic compounds. These structures can encapsulate therapeutics and be engineered to navigate biological barriers and deliver their payload to a disease site in a controlled, targeted fashion (38). Though a detailed analysis of nanoparticle technology is outside the scope of this review, the highly precise nature of nanoparticle-mediated drug delivery would synergize well with the targeted delivery capabilities offered by locoregional interventional approaches to immuno-oncology and merits brief discussion here.

In cancer immunotherapy, nanoparticles can be utilized to achieve site-specific effects and avoid systemic toxicity while circumventing the tumor microenvironment. In 2017, Schmid et al. demonstrated that immunomodulatory compounds delivered *via* T cell targeting nanoparticles achieved stronger T cell activation than systemic administration of the therapeutic in mouse models. Lipid nanoparticles containing a TGF-β inhibitor were synthesized with anti-CD8 antibodies conjugated to the particle surfaces and injected into mice expressing B16 melanoma, achieving high concentrations in tumor sites. The group further demonstrated the ability to specifically target tumor reactive lymphocytes by conjugating anti-PD-1 antibodies to the particle surfaces (39).

In another example, Gong et al. utilized pH-responsive nanoparticles for co-delivery of metformin with short interfering RNA (siRNA) against the fibrinogen-like protein (FGL1) gene; the nanoconstructs aimed to deliver metformin to block PD-L1 to prevent T cell suppression while simultaneously using siRNA against FGL1 to block another anti-inflammatory pathway. Of note, a pH trigger was utilized in these nanoparticles as the reaction of metformin with CO2 results in the low-pH-activated endosomal escape of the nanoparticle payload. *In vivo* studies were conducted in mice expressing breast cancer, and findings included significantly decreased tumor mass and increased survival in experimental groups (40).

These studies highlight the ability to engineer nanosystems for targeted delivery and demonstrate a clear potential for applications of nanoparticle technology in cancer immunotherapy. Basic science and clinical research in nanoparticles is highly active with many promising developments on the horizon. Magnetic biomaterials such as iron oxide nanoparticles are being explored for magnetic hyperthermia, a technique which oscillates nanoparticles in an alternating magnetic field to cause heat-induced death and DAMP generation of tumor tissue. Magnetic nanoparticles can also be engineered for highly precise targeting to tumor sites along with magnetic-mediated drug delivery and cellular uptake (41). CRISPR-Cas9, a potent tool for gene-editing, is another potential avenue for immunotherapy applications. These systems have well-documented applications in immunotherapy, ranging from CAR-T cell function augmentation to direct oncolytic activity (42). The challenge of low intracellular delivery of constructs can similarly be overcome with nanosystems. Cationic polymers and lipid nanoparticles have been demonstrated to have high *in vitro* and *in vivo* cellular penetration in preclinical studies, and clinical trials may soon be possible with these novel therapeutics (43). Technologies analogous to nanoparticles are already utilized in interventional radiology for cancer treatment approaches; for example, Selective Internal Radiation Therapy (SIRT) utilizes microspheres containing the radioactive isotope yttrium-90 to treat liver cancers and is favored in many circumstances due to its ability to achieve high therapeutic doses in tumor sites (44). Image-guided administration of nanoparticle-encapsulated immunotherapeutics similarly offers the potential for high concentrations within therapeutic sites in addition to their intrinsic localization properties.

## Conclusions

The field of interventional oncology as a whole is burgeoning with technological and clinical advancements, and many of the discipline’s most impactful discoveries are just starting to show their promise. Intratumoral administration of cancer immunotherapy in particular has significant potential to alter the landscape of cancer treatment in the coming years, both independently and in conjunction with conventional treatment strategies. Many treatment-resistant or difficult-to-reach malignancies could become accessible with further advances in locoregional therapeutic administration. Moreover, the exciting developments in biomaterials and biotechnology will further enhance the precision and efficacy of locoregional immunotherapy. It is important to keep in mind that many of these technologies are still being validated in preclinical models or early-stage clinical trials and are still several years away from seeing widespread therapeutic applications in humans. However, the promising results demonstrated by the various studies covered in this review and beyond signal high and rapidly growing interest and expectations for a revolutionary approach to cancer treatment from scientists, clinicians, and patients.

## Author contributions

All authors listed have made a substantial, direct, and intellectual contribution to the work and approved it for publication.
